# Huntington’s disease mouse models: unraveling the pathology caused by CAG repeat expansion

**DOI:** 10.12703/r/10-77

**Published:** 2021-10-21

**Authors:** Julia Kaye, Terry Reisine, Steve Finkbeiner

**Affiliations:** 1 Center for Systems and Therapeutics, Gladstone Institutes, San Francisco, CA, USA; 2 Independent Scientific Consultant, Santa Cruz, CA, USA; 3 Taube/Koret Center for Neurodegenerative Disease Research, Gladstone Institutes, San Francisco, CA, USA; 4 Department of Neurology and Physiology, University of California, San Francisco, CA, USA

**Keywords:** R6/2 mouse model, BACHD, Huntington’s disease, mouse models, neurodegeneration, neurodegenerative disease

## Abstract

Huntington’s disease (HD) is a neurodegenerative disease that results in motor and cognitive dysfunction, leading to early death. HD is caused by an expansion of CAG repeats in the huntingtin gene (*HTT*). Here, we review the mouse models of HD. They have been used extensively to better understand the molecular and cellular basis of disease pathogenesis as well as to provide non-human subjects to test the efficacy of potential therapeutics. The first and best-studied *in vivo* rodent model of HD is the R6/2 mouse, in which a transgene containing the promoter and exon 1 fragment of human *HTT* with 150 CAG repeats was inserted into the mouse genome. R6/2 mice express rapid, robust behavioral pathologies and display a number of degenerative abnormalities in neuronal populations most vulnerable in HD. The first conditional full-length mutant huntingtin (mHTT) mouse model of HD was the bacterial artificial chromosome (BAC) transgenic mouse model of HD (BACHD), which expresses human full-length m*HTT* with a mixture of 97 CAG-CAA repeats under the control of endogenous *HTT* regulatory machinery. It has been useful in identifying the role of mHTT in specific neuronal populations in degenerative processes. In the knock-in (KI) model of HD, the expanded human CAG repeats and human exon 1 are inserted into the mouse *Htt* locus, so a chimera of the full-length mouse protein with the N-terminal human portion is expressed. Many of aspects of the pathology and behavioral deficits in the KI model better mimic disease characteristics found in HD patients than other models. Accordingly, some have proposed that these mice may be preferable models of the disease over others. Indeed, as our understanding of HD advances, so will the design of animal models to test and develop HD therapies.

## Introduction

Huntington’s disease (HD) is caused by an expansion of CAG repeats in the huntingtin gene (*HTT*)^1^, which leads to neurological deficits, including motor impairment^2^ and cognitive decline^3^. Normal *HTT* alleles contain fewer than 35 CAG repeats, a CAG repeat of 40 or more is considered a fully penetrant mutation, whereas tracts of 36 to 39 CAGs impart an increased risk of developing the disease^4–6^. There is a well-established correlation between the number of CAG repeats and age of onset^7,8^. With extreme CAG expansion, symptoms develop in childhood, pathology is extensive, and life is short. However, CAG repeat length does not fully explain the severity of HD: 30% to 50% of the variation in age of onset is not related to CAG repeat length^9^. Polymorphisms in genes other than *HTT* contribute to age of onset in HD^9–12^. For example, polymorphisms in the FANC1-associated nuclease 1 (*FAN1*) gene, which encodes a DNA repair enzyme, affect age of onset of HD^11–13^.

In addition to motor and cognitive decline, neuropsychiatric symptoms, including depression and anxiety^14^, are present in patients with HD and are thought to typically predate the onset of motor symptoms^15^. Other common systemic features of HD include weight loss due to changes in metabolism^16^ and sleep and circadian rhythm disturbances^17^. Symptoms usually begin in midlife, and death follows within 10 to 20 years^18–20^.

A prominent neuropathological feature of HD is neurodegeneration, including neuronal death in the striatum, which is a major relay center of cortical signaling through the basal ganglia and is critically involved in regulating motor function and cognition^21,22^. As a consequence, impaired striatal physiology, including changes at glutamatergic, dopaminergic (DA), and cholinergic synapses, may be evident during the pre-symptomatic phase of HD^23–31^.

**Figure 1.**
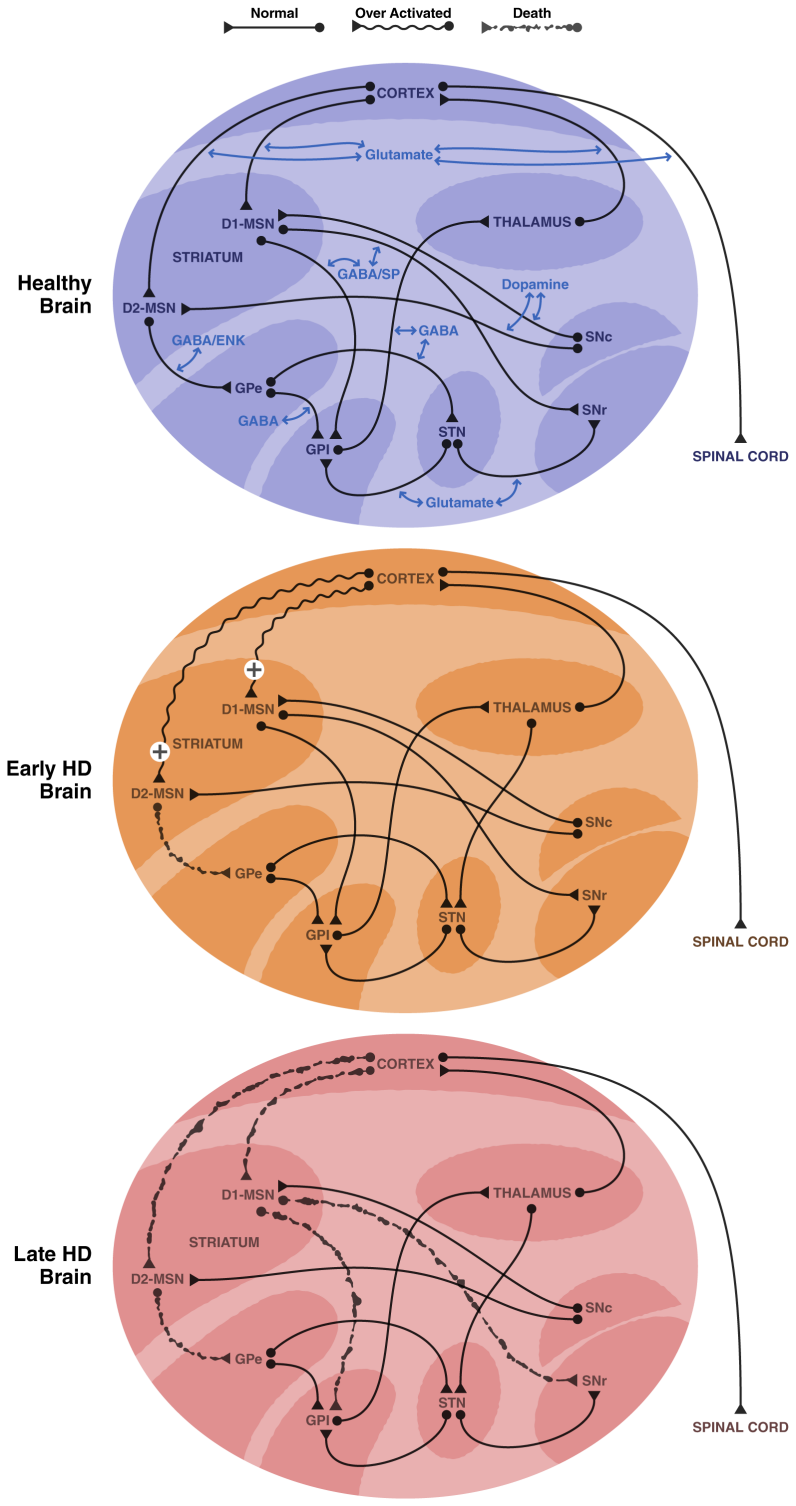
Alterations in Neuronal Circuitry in the Basal Ganglia in HD. **A**) Normal basal ganglia: The direct pathway consists of striatal medium spiny-like neurons (MSNs) expressing D1 dopamine receptors (D1-MSN) that project GABA/substance P (SP) neurons to the internal capsule of the internal globus pallidus (GPi) and the substantia nigra reticulata (SNr). The indirect pathway consists of MSNs expressing D2 dopamine receptors (D2-MSN) that project GABA/Enkephalin (Enk) neurons to the external capsule of the external globus pallidus (GPe) which then projects GABA neurons to the subthalamic nucelus (STN). The STN projects glutamate neurons to the GPi and SNr. The MSNs are innervated by dopamine neurons from the substantia nigra compacta (SNc) and by glutamate neurons from the cortex. The GPi projects inhibitory GABA neurons to the thalamus, and the thalamus projects excitatory glutamate neurons to the cortex. The motor cortex also projects glutamate neurons to the spinal cord to control movement. **B**) Basal ganglia at early stages of Huntington’s disease (HD): Cortical glutamate neuronal input to the striatum is hyperactive at early stages of HD, based on animal model work (R6/2 mice). This causes excitotoxicity. D2-MSNs are believed to be more sensitive to the glutamate hyperactivity and begin to degenerate first. The diminished GABA/Enk input to the GPe can produce an imbalance in the basal ganglia function and increase activity in the GPe which subsequently alters activity in the STN. **C**) Basal ganglia at later stages of HD: Both the cortex and striatum show degeneration in later stages of HD. The lost cortical glutamate neurons result in hypoactivity in the striatum. Both the D1-MSNs and the D2-MSNs degenerate.

Within the striatum, the most prominent neuropathology in HD is the loss of medium spiny-like neurons (MSNs), also known as spiny projection neurons (SPNs), and their cortical pyramidal neuronal innervation^2,32^. MSNs are the earliest affected neuronal population in HD (Figure 1) and undergo significant loss of dendritic structure and spines with disease progression in humans and animal models^33–39^. The cortico-striatal neurons are also affected in HD^40^ and impact cognitive decline^41^. The polyglutamine stretch in mutant huntingtin (mHTT) causes hyperactivity of glutamatergic cortico-striatal neurons and enhanced striatal glutamatergic transmission, which begins during the asymptomatic phase of HD^27–29,42^ and contributes to synaptic changes observed in later stages (Figure 1). Dysregulated glutamate release at cortico-striatal synapses results in aberrant calcium signaling leading to excitotoxity and is believed to be one of the causes of MSN vulnerability in HD^24,28,29^. Genetically reducing mHTT expression selectively in cortico-striatal neurons rescues electrophysiological alterations in striatal MSNs and reduces motor disabilities in mice^43^. Thus, early in the disease, a hyperactivity of glutamatergic cortico-striatal neurons driven by mHTT expression causes MSN dysfunction, which may influence the gradual death of striatal neurons over time. Brain imaging studies in pre-symptomatic HD carriers have shown that cortical atrophy occurs early, develops progressively, and correlates with the expression and severity of cognitive and motor symptoms^44–46^. The loss of cortico-striatal neurons leads to hypoglutamatergic input to the striatum at later stages of the disease. This suggests that the circuitry within the cortico-striatal glutamate neurons and MSNs plays a critical role in striatal dysfunction, MSN death, and HD pathogenesis^47^.

MSNs give rise to two distinct pathways that project to either the substantia nigra pars reticulata (SNr) and internal segment of the globus pallidus (GPi) (the direct pathway) or the external segment of the globus pallidus (GPe), which in turn projects to the subthalamic nucleus (STN)^48,49^ (the indirect pathway). MSNs projecting to the GPe appear to be affected earlier in HD than the other projection pathway and this has been proposed to cause an imbalance in the two pathways and the emergence of involuntary movements and chorea^50,51^. Whereas MSNs are vulnerable in HD, other striatal neuronal types such as GABA interneurons, including those expressing somatostatin and calretinin, are preserved in the disease^52^. It has been suggested that MSN degeneration may be linked to a loss of neurotrophic support^53,54^ and altered glutamate released from cortico-striatal neurons.

In addition to glutamatergic control, the different MSN projecting neurons are regulated by DA nigrostriatal neurons via activation of either D1 DA receptors or D2 DA receptors. The different populations of striatal MSNs differentially express the receptors; MSNs projecting to the SNr express D1 DA receptors, while MSNs projecting to the GPe primarily express D2 DA receptors^55,56^. Dopamine released from nigrostriatal neurons diminishes cortico-striatal glutamatergic excitation of the D2-expressing MSN striatopallidal neurons and may be neuroprotective^57–59^. In contrast, striatal stimulation of D1 DA receptors in MSNs appears to enhance glutamatergic transmission and may contribute to neurodegeneration and neuronal loss in the striatum and MSN-SNr projecting neurons^60–62^.

Whereas these studies suggest opposing roles of D1 and D2 DA receptors with regard to glutamate toxicity in the striatum, other work has shown that antagonism of both receptors reduces neuronal loss in the striatum^58^ and that blocking D2 receptor stimulation significantly reverses DA potentiation of mHTT-induced MSN cell death^63^. Consistent with the loss of MSNs in HD, both D1 and D2 DA receptor densities in the striatum are decreased in HD, even in asymptomatic patients, indicating that DA signaling is disrupted in the disease^64–66^. Striatal D1 and D2 DA receptor densities are also reduced in different animal models of HD^67–70^. Loss of DA receptors in patients with early-stage HD has been correlated with early cognitive decline^71^.

Genetic models have been developed to study both the molecular and cellular basis of HD and to provide systems to identify novel therapeutics, including small-molecule drugs, biologicals, nucleic acids, and cell-based therapeutics to slow progression of the disease. Here, we will focus on the development and use of transgenic mouse models to study HD. We emphasize electrophysical results that have begun to elucidate mechanisms of neuronal circuit dysfunction that may be related to behavioral deficits in HD.

## *In vivo* HTT transgenic mouse models

### R6/2 mouse model of Huntington’s disease

The first *in vivo* rodent model of HD to be developed was the R6 line, including the R6/1 and R6/2 models^72^. (See Table 1 for summary of results of mouse models.) The R6/2 mouse model, which is the most widely studied, has a transgene containing the promoter and exon 1 fragment of human *HTT* with 150 CAG repeats inserted into the mouse genome^73^. The R6/2 model robustly demonstrates the pathological hallmarks of HD, such as motor dysfunction and inclusion body (IB) formation and premature death^73–76^. The mice survive for about 15 weeks, and mHTT aggregates and inclusions form before the emergence of behavioral phenotypes, which include irregular gait, clasping, weight loss, increased grooming, and cognitive deficits by 5 weeks of age^73^. As the mice age, they develop seizures. Because of the rapid progression of disease phenotypes, the R6/2 mice have been proposed as a model of juvenile HD.

**Table 1.** Biology of transgenic mice models of Huntington’s disease.

Model	Genetic change	Behavioral phenotype	Pathology	Electrophysiology
R6/2^73^	Human exon 1 150 CAG repeats	Cognitive and motor deficits, irregular gait, clasping, weight loss^73–76^ Unique – seizures, early death (15 weeks)^73^	IB appear in neurons but death not clearly seen	Cortico-striatal hyperactivity^24,34,77–80,82–84,87–89^ leads to cortical neurodegeneration^57^ ↑ striatal D1R medium spiny-like neuron (MSN) activity^83^, ↓ striatal D2R MSN activity, ↑ dopaminergic neuroactivity^83^
N171-82Q^107^	cDNA encoding N-terminal human huntingtin (HTT) – 82 CAG repeats	Tremors, hypokinesia, lack of coordination, no weight gain, no seizures^108,109^	Striatal atrophy	Altered cortical and striatal connectivity seen with functional magnetic resonance imaging^110–116^
YAC^117,118^	Full-length human HTT 72/128 CAG repeats All regulatory sites present, similar level of mHTT found as wild-type HTT in controls^118^	Symptoms develop at 2–3 months: motor/cognitive deficits, hyperactivity, followed by difficulty in walking, followed by hypoactivity^104,117,119^	Striatal and cortical atrophy, selective loss of MSNs^104,117,119^	Similar to R6/2^24,28,80,88,89^
BACHD^120^	Conditional full-length 97 CAG/ CAA repeats	Progressive motor deficits start at 2–3 months, hypoactivity in open field, psychiatric symptoms, anxiety in light–dark box, depression–forced swimming^70,121,122^ Unique: weight gain	Striatal/cortical atrophy at 12 months, ↓ glutamate uptake and astrocyte phenotype, cortical degeneration	Synaptic pathology at 3 months^120,123^, cortical interneuronal and parvalbumin ↓ neuronal activity, results in ↑ cortico- striatal activity onto striatal MSNs related to reduced motor function^43,123^ Reducing mHTT in cortical neurons or MSNs improves synaptic dysfunction and motor functions^124^ Reduced mHTT in astrocytes improves behavior^125^
KI^126–130^	Human mutation in mouse gene CAG 140/175	Motor abnormalities, hyperactivity, repetitive movements at 1 month, followed by decreased activity, gait abnormalities subthalamic nucleus (STN) deficits occur prior to striatal dysfunction^130,131–134^	Loss of neurons by 2 years, also tyrosine hydroxylase (TH) loss, ↓ thalamic-striatal neurons, olfactory system dysfunction ↓ Spine density of MSNs^128,131–133,135–140^ By 12 months, 30% STN neurons die^141^	↑ GABA current in MSNs, ↓ excitatory post-synaptic currents in MSNs, loss of excitatory input to MSNs, autonomous, STN activity impaired^24,28,78,80–82,84,87,142–145^

BACHD, bacterial artificial chromosome (BAC) transgenic mouse model of Huntington’s disease; KI, knock-in mouse model with 140 or 175 CAG repeats; N171-82Q, N-terminal fragment of human mutant huntingtin (mHTT) of 171 amino acids and 82 CAG repeats; YAC, yeast artificial chromosome model with 72 or 128 CAG repeats.

R6/2 mice display a number of degenerative abnormalities in electrophysiological properties of MSNs and cortico-striatal neurons that may contribute to the rapid appearance and progression of motor and cognitive deficits. The MSNs of symptomatic R6/2 mice have reduced membrane capacitance and increased input resistance, caused by reduced K^+^ channel expression^24,34,77^. Spontaneous GABA currents and inhibitory post-synaptic currents are increased in R6/2 MSNs^78,79^, whereas excitatory post-synaptic currents (EPSCs) are decreased^24,80–82^, which together result in a decrease in burst firing in the striatum.

The electrophysiological properties of the two populations of striatal MSNs—those innervating the SNr and those innervating GPe—are differentially affected in R6/2 mice, resulting in an imbalance in striatal output that may contribute to motor dysfunction^83^. GABAergic MSNs that innervate the SNr have reduced activity in R6/2 mice, resulting in reduced GABA_A_ receptor–mediated responses in the SNr and hyperactivity of DA neurons. This hyperactivity may contribute to HD progression^57^. In contrast to the MSNs projecting to the SNr, GABAergic MSNs projecting to the GPe showed increased responses in the GPe. In addition, early on, the cortical pyramidal glutamatergic neurons that project to MSNs become hyperexcitable in R6/2 mice and this enhanced activity precedes behavioral symptoms^84^. This was shown in a study by Burgold *et al*. (2019)^85^, who used chronic *in vivo* two-photon calcium imaging to study neurons in the motor cortex of behaving R6/2 mice. The R6/2 mice showed hyperactivity of the cortical neurons prior to the appearance of motor deficits. Furthermore, Fernández-García *et al*. (2020)^86^ used optogenetic techniques to show that stimulation of degenerating secondary motor cortex neurons of R6/1 mice that project to the dorsolateral striatum reversed motor deficits and changes in long-term depression and normalized spine density within the striatum. The cortical neurons in the R6/2 mice exhibit decreased synchrony^87^, and synchrony between the cortex, striatum, and STN is disrupted^88,89^. In addition, glutamate uptake may be impaired^47^. Lack of uptake can lead to an overabundance of synaptic glutamate, which in turn can initiate an excitotoxic neuronal death cascade.

Relatedly, decreased expression of the glutamate transporter 1 (GLT-1) has been reported in the striatum and cortex of postmortem HD brains and R6/2 mice^90–96^. Studies in R6/2 mice showed that cortical pyramidal neurons are more sensitive to compromised glutamate reuptake and that failure in this system might lead to overactivation of glutamate receptors in the frontal cortex and striatum^91^. Interestingly, as frontal cortical pyramidal neurons in the R6/2 mice are more prone to paroxysmal activity, this brain area might be a trigger for the development of epileptic seizures observed in R6/2 mice^84,97^. GLT-1 is predominantly expressed in astrocytes^98^, suggesting a critical role of astrocytes in neurodegeneration, revealing that the pathology caused by mHTT is not limited to neurons^99,100^. The rapid progression of phenotypes in R6/2 mice is particularly useful in trying to identify potential disease-modifying agents. Indeed, recent studies using R6/2 mice have revealed a promising cell-based therapy to treat HD using human embryonic stem cell–derived neuronal stem cells (hNSCs)^101^.

Although the R6/2 model exhibits robust disease-related phenotypes, there are caveats. Primarily, the transgene contains only the first exon of *HTT*; therefore, the protein lacks motifs such as the HEAT domain, through which HTT interacts with other proteins^102,103^. In addition, this fragment lacks post-translational modification sites that control HTT cleavage and may contribute to toxicity of mHTT^104–106^. These limitations may explain why the pathological consequences of R6/2 mice differ from those of other transgenic mouse models of HD, described in more detail below.

### N171-82Q mouse model of Huntington’s disease

Another HD model that expresses an N-terminal fragment of human mHTT is the N171-82Q mouse. The mouse carries a cDNA encoding the N-terminus of human HTT with the initial 171 amino acids and 82 CAG repeats, driven by the mouse prion promoter^107^. N171-82Q mice show striatal atrophy and modest MSN degeneration, ventricular enlargement, and a failure to gain weight^108,109^. They develop tremors, hypokinesia, and lack of coordination. The N171–82Q mice have a more delayed disease onset and longer survival than R6/2 mice^107^.

N171–82Q mice also exhibit altered functional neuronal connectivity, as assessed by functional magnetic resonance imaging (fMRI)^110^. Bilateral connectivity between the motor cortices and somatosensory cortices is reduced in these mice, as is intrastriatal connectivity. The weak intrastriatal connectivity is positively correlated with striatal atrophy and reduced motor function. In these respects, the mice mirror patients with HD, as fMRI also shows altered functional connectivity of cortical and thalamic regions associated with impaired motor function in patients with HD^111–116^. Reduced intrinsic functional connectivity is present even in premanifest HD gene carriers and to a much larger extent in patients with manifest HD^111–116^. These studies suggest that mHTT causes disruption of normal neuronal and functional linkage of brain regions involved in motor control and cognition.

### YAC72 and YAC128 mouse models of Huntington’s disease

The first full-length human mHTT transgenic animal models harbored *HTT* with either 72 or 128 CAG repeats in a yeast artificial chromosome (YAC) that includes all of the human regulatory elements, such as the introns, integrated into the mouse^117,118^. These models displayed a less severe phenotype than the R6/2 mice and express mHTT at levels similar to endogenous HTT^118^. At about 2 or 3 months of age, the mice develop symptoms, including motor and cognitive deficits that correlate with the appearance of mHTT aggregates and striatal and cortical atrophy^104,117,119^. The progression of symptoms begins with hyperactivity, followed by difficulty in walking along a rotating rod, and then hypokinesia. Deficits in rotarod performance correlate with loss of striatal neurons^117^. Many of the electrophysiological abnormalities of the MSNs and cortico-striatal neurons observed in R6/2 mice are similarly found in the YAC128 mice^24,28,80^. There is also reduced synchrony between the cortex, striatum, and STN in the YAC128 mouse^88,89^. This is consistent with findings in HD brain showing a progressive disconnect between the cortex and striatum with progression of striatal degeneration^39,146^.

In 2015, Pancani *et al*. reported that a muscarinic M4 receptor drug reduced the excessive cortical glutamatergic transmission in cortico-striatal slices of YAC128 mice^147^. The normalization of glutamate transmission occurred via M4 receptors localized pre-synaptically to the cortical neuronal input. The drug also reduced motor deficits in the mice, suggesting that therapeutics designed to normalize the cortical striatal imbalance might be therapeutically useful.

Furthermore, Al-Gharaibeh *et al*. used the YAC128 model to demonstrate that induced pluripotent stem cell (iPSC)-derived NSCs have the potential as a treatment of HD^148^. Mouse iPSC-NSCs bilaterally implanted into the striatum of YAC128 mice differentiated into MSNs and reduced motor deficits. The protective effect of the cells was suggested to be related to their ability to increase levels of brain-derived neurotrophic factor, which supports the survival of remaining neurons in the striatum^149–151^.

### BACHD mouse model of Huntington’s disease

The first conditional full-length mHTT mouse model of HD was the bacterial artificial chromosome (BAC) transgenic mouse model of HD (BACHD), which expresses human full-length m*HTT* with a mixture of 97 CAG and CAA repeats under the control of endogenous *HTT* regulatory machinery^120^. In terms of phenotype, the BACHD model is similar to the YAC128 HD mouse in many ways; progressive motor deficits are apparent as early as 2 months of age, and striatal and cortical atrophy occur at 12 months^70^. BACHD mice display hypoactivity in the open-field test^121^ as well as changes in affective behavioral phenotypes such as increased anxiety and depressive behavior at 6 months^122^.

Many of these phenotypes parallel the development of electrophysiological deficits in cortical pyramidal neurons, cortical interneurons, and striatal MSNs. This progressive synaptic pathology occurs around 3 months of age, when the motor deficits are still mild^123^. Electrophysiological analysis of MSNs of 6-month-old BACHD mice demonstrated selective reduction of large-amplitude EPSCs in striatal neurons^120^. These changes are paralleled by decreased cortical parvalbumin (PV) interneuron excitation and decreased pyramidal cell inhibition, resulting in increased cortico-striatal excitability onto striatal MSNs and a decline in motor function^123^.

Because synaptic dysfunction in cortico-striatal neurons and striatal MSNs is a critical neurodegenerative process in HD, researchers have used the BACHD mouse to study the effect of mHTT deletion in cortical pyramidal neurons, striatal MSNs, or both^43^. BACHD mice show significant reductions in *N*-methyl-d-aspartate (NMDA)-evoked synaptic responses in striatal MSNs in slices, and genetically reducing mHTT levels in either cortical pyramidal neurons or MSNs partially reversed this deficit. Furthermore, the MSNs of BACHD mice show reduced activity, indicated by reduced spontaneous EPSCs and increased spontaneous inhibitory post-synaptic currents (IPSCs), and these deficits are ameliorated by reducing mHTT levels in cortical pyramidal neurons. The synaptic deficits were more effectively improved when mHTT was removed from both MSNs and cortical pyramidal neurons. In addition, reducing mHTT levels in cortical neurons also improved neuronal activity in cortical neurons^124^. These findings suggest that mHTT in both cortico-striatal pyramidal neurons and MSNs contributes to synaptic deficits in striatal MSNs.

Removal of mHTT from cortical pyramidal neurons or MSNs partially reversed motor behaviors measured in the rotarod and locomotion tests, but removal of mHTT from both neuronal populations was required to restore these motor behaviors to wild-type control levels^43^. BACHD mice also exhibit anxiety-like behaviors as measured by light–dark box exploration and depression-like behavior in a forced swimming test. Reducing mHTT in cortico-striatal pyramidal neurons or both cortico-striatal pyramidal neurons and MSNs significantly improved these psychiatric behavioral deficits, whereas mHTT reduction in MSNs alone did not.

These studies suggest that mHTT in striatal MSNs contributes to some aspects of striatal pathogenesis, but the pathogenesis of many behavioral and neurodegenerative phenotypes likely requires mHTT expression in other populations. In particular, dysfunction of cortical pyramidal neurons due to expression of mHTT contributes to synaptic deficits in MSNs and motor and psychiatric behavioral deficits. These findings indicate distinct but interacting roles of cortical and striatal mHTT in HD pathogenesis and support a role for non-cell-autonomous mHTT toxicity in striatal pathogenesis.

There is also significant evidence that non-neuronal populations contribute to HD^152^. The BACHD model was recently used to investigate the role of mHTT in astrocytes on disease phenotypes^125^. That study showed that selective reduction of mHTT in astrocytes in the cortex and striatum improved striatal MSN synaptic responses and behavioral phenotypes. mHTT in astrocytes may contribute to neuronal dysfunction by altering the regulation of extracellular glutamate and other key aspects of synaptic transmission. Altered glutamate release in HD models has been described both *in vitro* and *in vivo*^110,153^, and impaired glutamate signaling might further occur in HD as a consequence of decreased glutamate uptake. Studies of HD postmortem brain and HD transgenic models have consistently shown decreased GLT-1, which is responsible for the bulk of glutamate uptake in astrocytes^93–95^. These findings support the role of astrocytes in mHTT-induced HD pathophysiology.

BACHD mice have also been used to study the role of post-translational modifications of mHTT, in particular protein phosphorylation, in disease pathogenesis. Three potential phosphorylation sites—serines 13 and 16 in the N-terminal region and serine 421 in the Akt consensus sequence—were studied^154,155^. Biochemical studies have shown that phosphorylation at serines 13 and 16 significantly alters the structure of mHTT^155^, and phosphorylation at serine 421 alters the transport of mHTT^156^, suggesting that post-translational modifications at these sites may affect function of this protein. For the studies in BACHD mice, mHTT constructs were generated in which the serines were converted to aspartates to mimic phosphorylation or to alanine to prevent phosphorylation. The phosphomimetic substitutions were protective and rescued locomotor deficits and anxiety-like behaviors and reduced striatal neuronal loss, whereas the alanine mutations did not hinder the pathogenic actions of mHTT. In the case of the serine 421 site, the phosphomimetic substitution reduced steady-state levels of pathogenic soluble mHTT and increased turnover to improve clearance of mHTT. These studies thus identified specific structural changes in mHTT that may be responsible for pathophysiology in HD.

### Knock-in Huntington’s disease mouse models

Knock-in (KI) mouse models of HD consist of the human HD mutation inserted into the mouse *HTT* gene locus. Because the mutation is expressed in its appropriate genomic and protein context, these models are believed to more accurately represent the genetic basis of HD and have been used extensively to investigate the pathophysiology of HD and potential treatments^126^. The three KI models that have been studied the most are the Q140, Hdh(CAG)150, and Q175 mice, although other models with varying CAG expansions, including those with 50, 92, and 111 CAG repeats, have also been developed and studied^157,158^. In the Q140 mice, mouse exon 1 of *HTT* is replaced by a mutated version of human exon 1^127,128^. Robust behavioral deficits as well as motor abnormalities have been detected in homozygous CAG140 mice^128,135,136^. The neuropathology consists of mHTT nuclear staining and aggregates in the striatum and cortex, which become intense and widespread at only 4 months of age. The early pathology corresponds to brain regions that receive DA inputs, supporting the relationship between dopamine and HD pathology^128,159^. mHTT aggregates are also first seen in the striosomes, consistent with the early vulnerability of this region in humans^137^. These mice also show early loss of thalamic-striatal neuronal input to MSNs, which may contribute to striatal dysfunction manifest as diminished excitatory drive in the striatum^138^. In old age (1–2 years), CAG140 mice show late striatal neuronal loss and atrophy. Surviving neurons express loss of spines and reduced dendritic complexity. The olfactory system displays early and marked aggregate accumulation, which may be relevant to the early deficit in odor discrimination observed in patients with HD^139,140^.

In the Hdh(CAG)150 model, 150 CAG repeats were inserted into the mouse *HTT* but no human sequences are included^129^. This model shows a delayed onset of symptoms compared with CAG140 mice but exhibit motor defects, such as balance and gait abnormalities, as well as clasping and weight loss, by 40 weeks of age^131^. Cognitive deficits occur at around 24 weeks of age^132^. By 22 months, they show widespread mHTT aggregation throughout the brain and transcriptional dysregulation^160^.

Interestingly, a study by Arnoux *et al*. (2018)^161^ using *in vivo* two-photon Ca^2+^ imaging in premanifest Hdh(CAG)150 KI mice showed increased neuronal activity in the visual cortex. This finding is consistent with early signs of hyperactivity in cortical networks found in other HD models and the finding that early in HD the visual cortex is one of the first brain regions to show dysfunction^162^.

Side-by-side comparison of CAG140 and Hdh(CAG)150 mice by Franich *et al*. (2019)^133^ showed that CAG140 mice exhibit earlier onset of behavioral deficits and formation of nuclear inclusions. The authors proposed that these differences may be due to an incompletely spliced *HTT* exon 1 transcript in the CAG140 mouse, which encodes the highly pathogenic exon 1 mHTT protein^163,164^ which leads to early aggregation. The very early phenotypic deficits in the Q140 mice have made them an ideal model for testing novel therapeutic interventions.

The Q175 KI mouse is a spontaneous extension of the Q140 KI^130^. The Q175 KI mouse shows behavioral changes, including motor, cognitive, and circadian deficits^130^. These mice exhibit gait abnormalities at 4 weeks of age, hypoactivity as measured in the open-field test by 4 months^130,134^, rotarod and climbing abnormalities at 30 weeks of age, and cognitive deficits at 12 months. mHTT aggregates are widely distributed throughout the brain, and the number of neurons containing nuclear inclusions increases with age in both the striatum and cortex^134^. Morphological alterations include decreased numbers of MSNs and striatal volume loss^81^.

Both CAG140 and Q175 mice have been used to study synaptic changes caused by mHTT. Within the striatum, there is a decrease in burst firing in CAG140 KI mice^142,143^, consistent with decreases in EPSCs and increases in IPSCs, much like those found in R6/2 mice^24,78^. The cortex of CAG140 mice shows increased EPSC frequency^84^ and decreased synchrony^87^. Donzis *et al*. (2020)^165^ used two-photon laser-scanning microscopy on symptomatic Q175 mice to study network circuitry in the motor cortical neurons and found that calcium transients had reduced amplitude, suggesting decreased bursting activity. In contrast, in pre-symptomatic Q175 mice, neuronal activity was increased, consistent with a switch in activity of these neurons over time.

Electrophysiological studies have shown that spontaneous GABAergic currents in striatal MSNs are increased in symptomatic Q175 mice^78^ but that EPSCs in MSNs are decreased^24,80–82^ because of alterations in glutamatergic inputs from the cortex and thalamus^28,78,144^. Significant decreases in spine density of MSNs were found in Q175 mice. The increase in frequency of IPSCs combined with the decrease in frequency of EPSCs generate an imbalance in the ratio of inhibition to excitation, which is relevant for understanding phenotype progression.

This notion is supported by evidence that shows a decline in the glutamate-to-GABA ratio measured by high-performance liquid chromatography in 6-month-old Q175 mice^141^. Striatal interneurons—both persistent, low threshold-spiking somatostatin-expressing interneurons and fast-firing PV-expressing interneurons—are principal sources of the rise in inhibition seen in MSNs in the R6/2 and BACHD models^166^. Loss of excitatory inputs to MSNs, which seem to be associated with loss of dendritic spines and increased inhibitory inputs to MSNs, is exhibited by the Q175 mice.

Studies in R6/2 mice showed that in addition to an alteration of striatal MSN properties, there were changes in output regions of MSNs that may contribute to the pathophysiology of HD. Similarly, Atherton *et al*. showed that in Q175 mice, STN neurons have altered synaptic properties indicative of dysfunction and degeneration^145^. The STN is a critical component of the direct and indirect MSNs output pathways and is critical for constraining cortico-striatal activity underlying action selection^48,167^. In Q175 mice, autonomous STN activity is impaired because of activation of K^ATP^ channels. STN neurons exhibit prolonged NMDA receptor–mediated synaptic currents due to deficient glutamate uptake, which can be rescued by NMDA receptor antagonism. At 12 months of age, about 30% of STN neurons are lost in these mice^141^. The STN dysfunction and neuronal loss precede striatal cell death and cortico-striatal abnormalities and occur prior to the onset of major behavioral symptoms. Thus, dysfunction and degeneration of cortical and striatal neurons occur in concert with profound changes in other elements of the basal ganglia. Dysfunction within the STN is an early HD feature that may contribute to its expression and course^145^.

Interestingly, the availability of KI mouse models with a large range of CAG repeats (50, 92, 111, 140, 150, and 175 CAG repeats) has facilitated studies to establish the relationship between CAG repeat length and the changes in behavior and brain transcription that are linked to the progression of pathogenesis^168,169^. Similarly, Ament *et al*. (2017)^170^ used KI mice with different CAG repeats to begin to understand the molecular basis of CAG repeat instability in the striatum linked to neurodegeneration. Others have also used the KI mice to investigate the role of epigenetic changes that contribute to chromatin remodeling^171^ and DNA repair alterations in HD^172,173^.

## Conclusions

The development of *in vivo* animal models of HD has greatly added to our understanding of the biology of HD and the molecular and cellular pathways that drive pathogenesis. One issue to consider in the different mouse models is the different forms of mHTT expressed in each. Those with fragments, such as the R6/2 mouse, may produce exaggerated phenotypes and, because the expressed protein lacks downstream regulatory sites, may lack the full range of mutant *HTT* gene and protein–protein interactions. Although the BACHD model has provided important information on structural aspects of the mHTT protein that impact disease behaviors such as locomotion and anxiety, this model is unusual in that the mice gain excessive weight whereas most patients with HD generally have greatly reduced weight. Similarly, the YAC128 mice which express full-length human mHTT also exhibit weight gain. This anomaly may be unrelated to CAG repeat length and has been proposed to be due to the impact of HTT on the expression of IgF-1^174^. In the KI model, the expanded human CAG repeats and human exon 1 are inserted into the mouse *Htt* locus, so a chimera of the full-length mouse protein with the N-terminal human portion is expressed. Many aspects of the pathology and behavioral deficits in the Q140 KI mouse and the spontaneously expanded Q175 KI mouse mimic disease characteristics found in patients with HD, and importantly those phenotypes are robust. Accordingly, some have proposed that these mice may be preferable models of the disease^133^.

The best model is the one that is the most predictive of human disease. Unfortunately, animal models for most human neurodegenerative diseases have historically been poor at predicting which therapeutics are most likely to work in humans. However, as our understanding of the disease mechanisms of HD advances, so will the design of animal models to discover and test innovative therapeutics that may be translated into treatment to slow the onset and progression of HD.
